# Supplementary material for: Empirical methods for controlling false positives and estimating confidence in ChIP-Seq peaks
Source: BMC Bioinformatics. 2008 Dec 5;9:523. doi: 10.1186/1471-2105-9-523 (PMC2628906; doi:10.1186/1471-2105-9-523)
Supplement: Additional File 1 — A variety of html documents from the USeq web site detailing the available applications, their best usage, output file type descriptions, command line menus, etc. [file 1471-2105-9-523-S1.zip › USeqUserGuides/JavaDoc.zip!/JavaDoc/trans/graphics/GenomicRegionGlyph.html]

GenomicRegionGlyph


|  |  |  |
| --- | --- | --- |
| **Overview** \| **Package** \| **Class** \| **Use** \| **Tree** \| **Deprecated** \| **Index** \| **Help** | |  |
| **PREV CLASS**   **NEXT CLASS** | **FRAMES**    **NO FRAMES**     **All Classes** |  |
| SUMMARY: NESTED \| FIELD \| CONSTR \| METHOD | DETAIL: FIELD \| CONSTR \| METHOD |  |


---


## trans.graphics Class GenomicRegionGlyph

```
java.lang.Object
  trans.graphics.GenomicRegionGlyph
```

---

``` public class GenomicRegionGlyph extends java.lang.Object ```

---

**Constructor Summary**

`GenomicRegionGlyph(java.lang.String label,
int bpLength)`


| **Method Summary** | |
| --- | --- |
| `void` | `draw(java.awt.Graphics2D g2, java.awt.font.FontRenderContext fontContext, java.awt.Font font)` |
| `java.lang.String` | `getLabel()` |
| `java.awt.geom.Point2D` | `getPoint()` |
| `java.awt.geom.Rectangle2D` | `getRectangle()` |
| `void` | `makeRectangleAndPoint(boolean rightSide, double topXCoor, double topYCoor, double widthScalar, double height, java.awt.Color rectangleColor)` |

**Methods inherited from class java.lang.Object**

`equals, getClass, hashCode, notify, notifyAll, toString, wait, wait, wait`

**Constructor Detail**

### GenomicRegionGlyph

```
public GenomicRegionGlyph(java.lang.String label,
                          int bpLength)
```


**Method Detail**

### makeRectangleAndPoint

```
public void makeRectangleAndPoint(boolean rightSide,
                                  double topXCoor,
                                  double topYCoor,
                                  double widthScalar,
                                  double height,
                                  java.awt.Color rectangleColor)
```

---


### draw

```
public void draw(java.awt.Graphics2D g2,
                 java.awt.font.FontRenderContext fontContext,
                 java.awt.Font font)
```

---


### getPoint

```
public java.awt.geom.Point2D getPoint()
```

---


### getRectangle

```
public java.awt.geom.Rectangle2D getRectangle()
```

---


### getLabel

```
public java.lang.String getLabel()
```


---


|  |  |  |
| --- | --- | --- |
| **Overview** \| **Package** \| **Class** \| **Use** \| **Tree** \| **Deprecated** \| **Index** \| **Help** | |  |
| **PREV CLASS**   **NEXT CLASS** | **FRAMES**    **NO FRAMES**     **All Classes** |  |
| SUMMARY: NESTED \| FIELD \| CONSTR \| METHOD | DETAIL: FIELD \| CONSTR \| METHOD |  |


---
